# Contactless Rotor Ground Fault Detection Method for Brushless Synchronous Machines Based on an AC/DC Rotating Current Sensor

**DOI:** 10.3390/s23229065

**Published:** 2023-11-09

**Authors:** Miguel A. Pardo-Vicente, José M. Guerrero, Carlos A. Platero, José A. Sánchez-Férnandez

**Affiliations:** 1 Hydraulic, Energy and Environmental Engineering Department, Universidad Politécnica de Madrid, 28040 Madrid, Spain; ma.pardo@alumnos.upm.es (M.A.P.-V.); joseangel.sanchez@upm.es (J.A.S.-F.); 2 Electric Department, Universidad del País Vasco/Euskal Herriko Unibertsitatea, 48013 Bilbao, Spain; josemanuel.guerrerog@ehu.eus; 3 Automatic, Electric and Electronic Engineering and Industrial Computing Department, Universidad Politécnica de Madrid, 28003 Madrid, Spain

**Keywords:** alternate current, direct current, rotor fault detection, current sensor

## Abstract

Brushless synchronous machines (BSMs) are replacing conventional synchronous machines with static excitation in generation facilities due to the absence of sparking and lower maintenance. However, this excitation system makes measuring electric parameters in the rotor challenging. It is highly difficult to detect ground faults, which are the most common type of electrical fault in electric machines. In this paper, a ground fault detection method for BSMs is proposed. It is based on an inductive AC/DC rotating current sensor installed in the shaft. In the case of a ground fault in the rotating parts of the BSM, a fault current will flow through the rotor’s sensor, inducing voltage in its stator. By analyzing the frequency components of the induced voltage, the detection of a ground fault in the rotating elements is possible. The ground faults detection method proposed covers the whole rotor and discerns between DC and AC sides. This method does not need any additional power source, slip ring, or brush, which is an important advantage in comparison with the existing methods. To corroborate the detection method, experimental tests have been performed using a prototype of this sensor connected to laboratory synchronous machines, achieving satisfactory results.

## 1. Introduction

**Figure 1:**
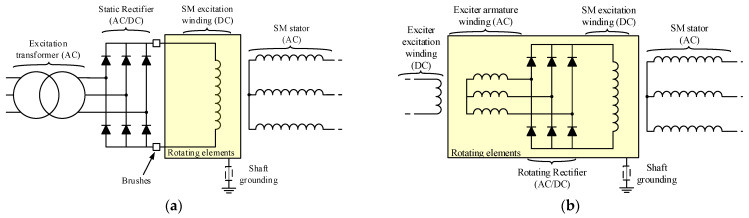
Electrical schemes of synchronous machines [(**a**): static excitation SM electrical scheme; (**b**): BSM electrical scheme].

Brushless synchronous machines (BSMs) are widely implemented in electricity generation power plants [1,2], high-efficiency processes [3], and systems where sparking should not happen, such as in aircraft applications [4,5]. The absence of brushes to feed the excitation winding of the main synchronous machine (SM) implies a second machine, called an exciter. The exciter is a synchronous machine whose armature winding is in its rotor and its field winding is in its stator. The stator of the exciter is fed from a static excitation system. The exciter armature winding is connected to the field winding of the main SM through a rotating rectifier. Therefore, the exciter armature currents are transformed into DC for the excitation of the main SM. The differences between a BSM and a static excitation SM can be observed in Figure 1.

Despite BSMs’ high efficiency and large operating lifetime, the fault diagnosis in these machines is challenging. This is due to the lack of rotor electrical measurements, as there are not many sensors suitable for rotating operation. In a previous research work [6], the authors developed an AC/DC current sensor based on an inductive coupling between two windings placed in the stator and rotor, respectively.

The development of novel techniques for current measurement is a very active research topic. For example, in the case of load monitoring [7], where the current wave form is very important, in the case of distortion in power systems [8], or even in the case of lightning current measurement [9] using optical sensors.

The absence of brushes in BSMs does not allow for obtaining measurements of rotor electrical magnitudes. Rotor faults account for around 10% of total machine electrical and mechanical faults [10], and their detection in BSMs is not an easy task.

Different types of faults can appear in the rotor of BSMs, such as diode faults [11], inter-turns faults, or phase-to-phase faults [2], broken wires or open phase faults [12], and ground faults (GFs) [13,14]. There are well-known commercial rotor ground fault protection relays based on injection methods by AC or DC injection sources. Their main drawback is the need for auxiliary brushes and sliprings. For this reason, these methods cannot be used in applications where the absence of sparking is required. Another technique, proposed by this research team, is using the exciter as an injection source. However, this technique requires, also, the installation of a slipring and brush [15]. Therefore, the sparking problem remains.

Ground faults, mainly caused by insulation aging, are the most common type of faults in power systems [16] and one of the most common in electric machines. In ungrounded systems, at first, a GF does not provoke significant alterations in the circuit. However, if this fault is not repaired and a second one occurs, a large fault current will flow through the grounded points, causing severe damage in the machine. Therefore, protecting the machines against ground faults is very important.

On the one hand, machine stator protection against GFs can be achieved by installing differential relays [17]. However, this application is effective only for low-impedance grounding. It is a common practice in power generation applications to overcome the limitation of ground fault currents using high-impedance grounding. Therefore, the differential relays do not see GFs. In this case, zero sequence voltage protections are recommended [18]. Another possibility is injecting an AC current between the ground and the stator winding [19,20], which also outlines the fault location. But these latter methods are complex to implement. As an additional solution, 100% earth–stator protections can be used by combining a neutral overvoltage protection with a third harmonic minimum voltage protection [21].

**Figure 2:**
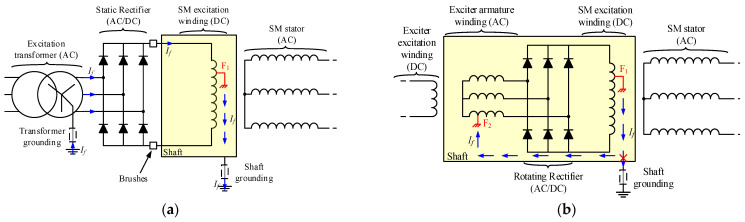
Examples of ground fault current paths for different types of synchronous machines [(**a**): static excitation SM ground fault current path through excitation transformer grounding impedance; (**b**): BSM ground fault current path, F1: first ground fault, F2: second ground fault].

On the other hand, the field windings of SMs with static excitation can be protected using active methods that inject an AC or DC current between the terminals of the winding and ground [22]. The injection device can easily be placed in the static side of the excitation system. However, the shaft must be connected to the ground through an additional brush or a cooper braid. In this case, the fault current will flow through the braid to the AC or DC voltage injection source and the current can be measured. In [23,24], the AC or DC injection source is not required, as the secondary of the excitation system feeding transformer is grounded through a high-value resistor that limits the maximum fault current. With the same phenomenon, the fault current flows through the grounding resistor. If the voltage between the terminals of this resistor is different from zero, it implies a GF in the excitation system. The fault current path of this system, between the GF and its grounding, can be observed in Figure 2a. It corresponds to a static excitation synchronous machine where the neutral of the excitation transformer has been grounded through a limiting resistor. Other off-line detection methods can also be implemented once the machine has previously tripped or during maintenance, for example, frequency response analysis (FRA) methods. However, these FRA methods need, in most cases, to extract the rotor to perform the diagnosis.

Nevertheless, in brushless applications, even if the shaft is grounded, the fault current will not flow outside of the rotor as the returning path is opened between the groundings of the static parts (exciter excitation winding) and the rotating parts. Consequently, the fault current will not flow outside of the rotor and the shaft. This fact makes challenging the GF detection with relays placed out of the rotor, and, consequently, most of the BSM protection relays are based on the interturn or rotor phase short-circuit faults, i.e., the first GF is not detected, and the protection relays wait for a second GF that provokes a more severe situation. This last concept can be seen in Figure 2b, which represents a double ground fault in the excitation circuit of a BSM. Common techniques to detect the short circuits in the rotor of BSMs are airgap flux monitoring [12,25], stray flux monitoring [26], or static electrical parameters analysis [2,12]. In the case of flux monitoring, airgap flux techniques are more invasive than stray flux ones, but they provide higher precision when measuring. The interturn faults are detected due to little asymmetries in the flux measurements because the short field winding is able to create less flux than healthy ones [25,26]. In [2], the state of the exciter armature winding is monitored based on the relative percentage of the first and second harmonics of the stator field current compared with its DC component. If this percentage is over a certain threshold, it implies the existence of a fault in the rotor. The study presented in [12] also proposes the stator current measurement, since a failure in the rotor provokes alterations in the airgap flux which, at the same time, are reflected in the output currents. Finally, [26] analyzes the transient exciter and SM stator currents at the same time that the airgap and stray flux. Attending to the time-frequency plot of each parameter, field winding inter-turns faults of the main machine can be diagnosed.

In this paper, the monitoring of rotors in BSMs against ground faults, based on the use of an AC/DC rotating current sensor, is proposed. Consequently, the problem of waiting until a short circuit is produced by two GFs is avoided, and an early diagnostic of the BSM can be achieved. This method is based on measuring the leakage current through the neutral of the power supply and ground in the case of fault.

A previous method was developed for static excitation systems [23]. In that case, the neutral of the excitation transformer was grounded by a limiting resistor (see Figure 2a). In the case of a fault in the AC side of the excitation, the leakage current frequency corresponds to the grid frequency (*f*_1_). On the other hand, if the fault is in the rotor, the frequency is three times the grid frequency (3 × *f*_1_) [24].

Afterwards, this previous method was adapted and used for brushless excitation systems [15]. In this case, the armature winding of the exciter should be in star connection with neutral. Also, it was necessary to install an auxiliary slip ring and brush on the neutral of the exciter. A limiting resistor was connected from the auxiliary brush to ground in order to measure the leakage current.

To eliminate the auxiliary slip ring and brush, a rotating AC/DC current sensor was previously developed. The sensor has two windings: rotor and stator. The induced voltage in the sensor’s stator winding is proportional to the current flowing through the sensor rotor winding. The sensor was designed and tested by injecting AC and DC currents in order to obtain the V/mA ratio [6], but its behavior in BSMs was not validated.

The main contribution of this paper is the validation of the complete system and a brushless synchronous machine with the neutral of the exciter armature connected to the ground through the rotating AC/DC current sensor via experimental tests in healthy and faulty conditions. Despite the fact that there are several factors that affect the operation of the system, such as the impedances of the sensor for different frequencies, rotor capacities to the ground, or electromagnetic noise, the results have been satisfactory.

The advantages of the proposed method are mainly as follows:

- It allows for the detection of a first ground fault in BSMs before a short-circuit appears, avoiding a high level of damage in the machine and providing an early diagnostic of the machine.
- It does not need any brush to measure the ground fault current in the rotor, so possible sparking problems are avoided.
- It can discern if the fault is taking place in the armature winding of the exciter or in the field winding of the main SM attending to the frequency components of the sensor’s measured voltage.

The remainder of this paper is organized as follows: Section 2 explains in detail the method’s operating principles and sensor characteristics. Then, Section 3 describes the experimental setup, and Section 4 shows the results obtained. Section 5 discusses these results, and finally, Section 6 concludes the paper, highlighting the main ideas of the manuscript.

## 2. Operation Principles of the Rotor Ground Fault Detection Method

The proposed method requires a rotating AC/DC current sensor coupled in the rotating shaft of the main BSM and the exciter. The current sensor is able to measure rotor AC and DC currents, analyzing the voltage induced in its stator. This section is focused on two parts, a brief description of the sensor and then the description of the detection method.

### 2.1. Inductive AC/DC Rotating Current Sensor Brief Description

**Figure 3:**
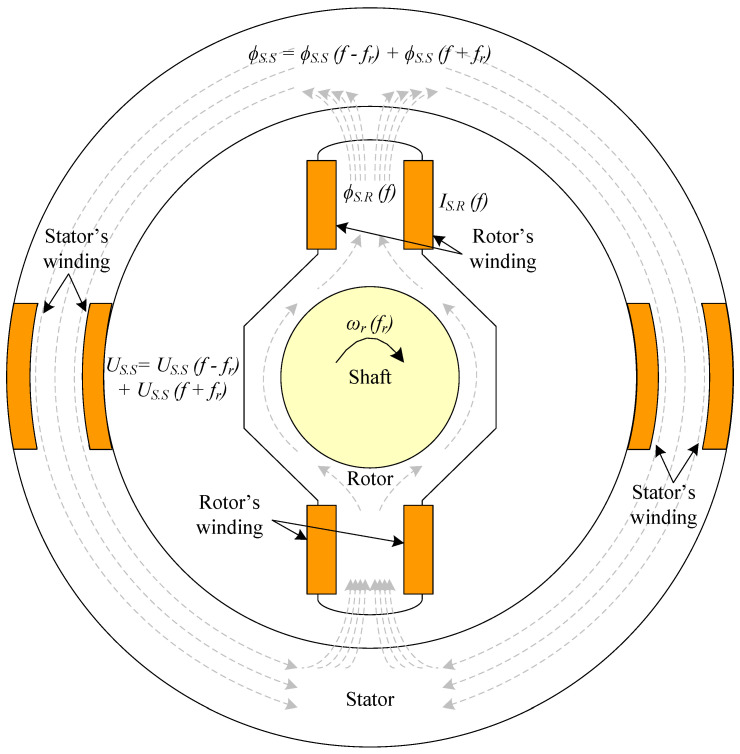
Current sensor coupled in the BSM shaft for the proposed contactless online ground fault detection.

This subsection briefly describes the sensor which was previously developed by the authors. A detailed description of its design, operation, accuracy, and performance can be found in [6]. The calibration for DC and 450 Hz AC current measurements is also included. The sensor has a rotating part coupled to the rotating shaft of the machine that will be diagnosed. The reliability of the sensor is similar to that of any rotating machine, and its cost is negligible compared to the cost of a large brushless generator. It has two windings in series, conforming a pair of poles. The stator also has two windings connected in series, located only in a concentrated zone. The sensor’s radial section can be seen in Figure 3.

In Figure 3, theorical fluxes are also plotted. First, the winding placed in the rotor measures a certain current *I_S.R_* with a frequency, *f*. This current will generate a flux, *ϕ_S.R_*_,_ in the rotor of the sensor with the same frequency of this *I_S.R_* current. Following the magnetic circuit, *ϕ_S.R_* passes through the airgap to the stator of the sensor. According to the Leblanc theorem, the flux is decomposed in two components: one with the frequency of *ϕ_S.R_* plus the rotor’s frequency (*f* + *f_r_*), and the other with the frequency of *ϕ_S.R_* minus the rotor’s frequency (*f* − *f_r_*). According to (1), the flux in the stator, *ϕ_S.S_*, is then:

(1) $$\phi_{S.S}=\phi_{S.S}\left(f+f_{r}\right)+\phi_{S.S}\left(f-f_{r}\right)$$

Afterwards, the voltage in the stator winding of the sensor, *U_S_*, can be monitored in real time, indirectly measuring the flux variations as:

(2) $$U_{S}=N\cdot \left\{\frac{d}{dt}\left[\phi_{S.S}\left(f+f_{r}\right)\right]+\frac{d}{dt}\left[\phi_{S.S}\left(f-f_{r}\right)\right]\right\}$$

where *U_S_* will have the same frequency components as *ϕ_S.S_*. From (2), it can be seen that the voltage is proportional to the number of turns of the sensor stator winding. Thus, as resolution increases with increasing voltage, it rises as the number of stator winding turns increases.

### 2.2. Ground Fault Detection Method Description

**Figure 4:**
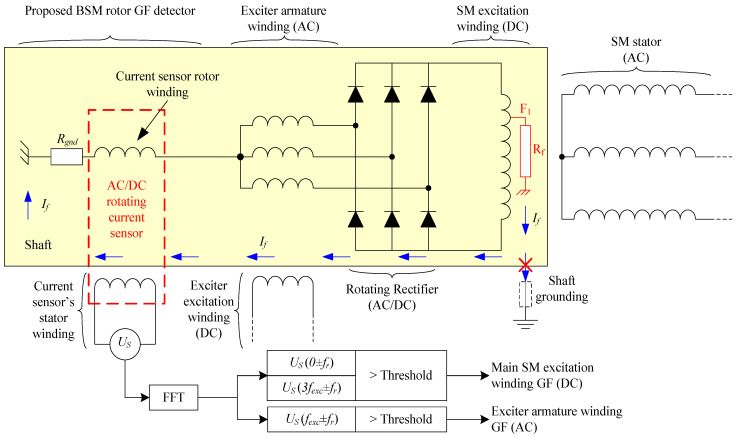
Proposed ground fault detection method for BSMs based on a rotating current sensor connected between the neutral of the armature winding of the exciter and the shaft’s chassis.

Once able to measure the current in the rotor, indirectly by the analysis of the voltage induced in the sensor’s stator, the GF detection method for BSMs is carried out by installing the sensor according to Figure 4. In this figure, the neutral of the armature winding is connected in series with one terminal of the sensor’s rotor. The other terminal is connected directly to the shaft. If needed, a resistive current limiter, *R_gnd_*, can be connected between the other rotor terminal and the shaft.

With this configuration, ground faults can be detected in the whole rotating elements, i.e., in the armature windings of the exciter (AC side) or in the field winding of the main machine (DC side).

In both cases, even if the shaft is grounded, the fault current path is closed inside the shaft and also limited by *R_gnd_* to low values. Furthermore, the fault current, *I_f_*, will be measured in the rotor’s sensor (*I_S.R_* = *I_f_*).

#### 2.2.1. AC Side: Armature Winding of the Exciter GF Detection

On the one hand, for a fault in the armature winding of the exciter, the fault current will have the frequency of the exciter AC side, which depends on the shaft rotating frequency and the number of pole pairs of the exciter machine. Therefore, *I_R.S_* will have the frequency of the exciter, *f_exc_*, and also the rotor’s flux, *ϕ_S.R_*. However, as explained before, due to the Leblanc theorem, *U_S_* will measure the other harmonic components that, in this case, correspond with the frequencies: *f_exc_* + *f_r_* and *f_exc_* − *f_r_*.

#### 2.2.2. DC Side: Field Winding of the Main Machine GF Detection

On the other hand, for faults in the main machine field winding (an example can be seen in Figure 4), the *I_f_* has mainly a DC and 3·*f_exc_* components. The DC component depends on the fault position, and the 3·*f_exc_* component depends on the fault resistance [24]. The DC flux induces voltages at the rotor frequency. And the 3·*f_exc_* component induces 3·*f_exc_* + *f_r_* and 3·*f_exc_* − *f_r_* in the stator of the current sensor. In this research work, a simple voltage threshold is proposed for detection purposes. However, a more complex analysis will be performed in a future research work with the representative harmonics of *U_S_* obtained by Fast Fourier Transform (FFT).

Additionally, as the current sensor can be connected to the shaft with a high value *R_gnd_*, the current is limited and the BSM does not have damages, even in the case of an operation with a ground fault. This fact allows us to acquire a huge number of *U_S_* wave cycles to improve the FFT process without damaging the machine.

**Figure 5:**
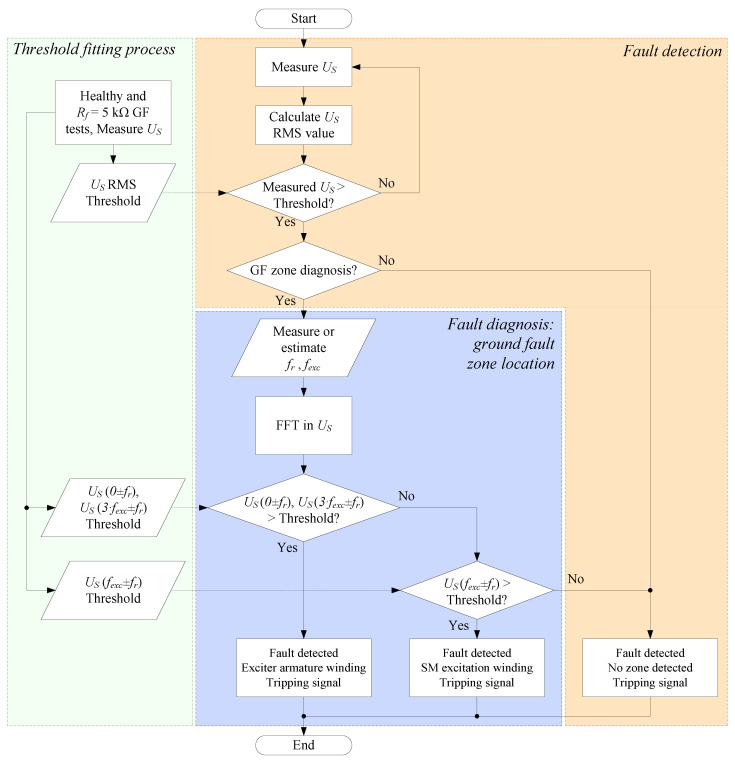
Flowchart of the proposed method for ground fault detection and diagnosis of BSM rotors.

Finally, a flowchart of the diagnosis method has been plotted in Figure 5 summing up the entire process. The detection process can be performed with only RMS evaluation in the stator terminals of the AC/DC current sensor; meanwhile, for the GF zone location, FFT should be carried out. The threshold can be set from the healthy operation and a ground fault with *R_f_* = 5 kΩ, which is the standard value for ground fault protection setting.

## 3. Experimental Setup

**Figure 6:**
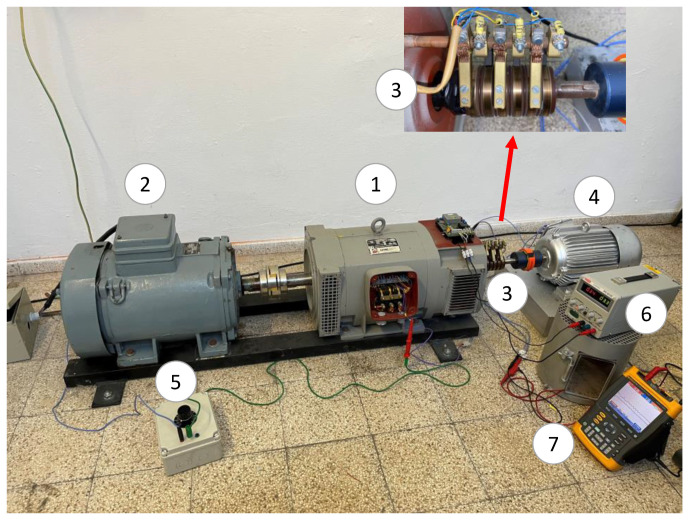
Experimental setup [(1): brushless synchronous machine; (2): induction motor; (3): brushes and slip rings; (4): rotating current sensor; (5): variable fault resistance; (6): adjustable voltage source; (7): oscilloscope].

**Figure 7:**
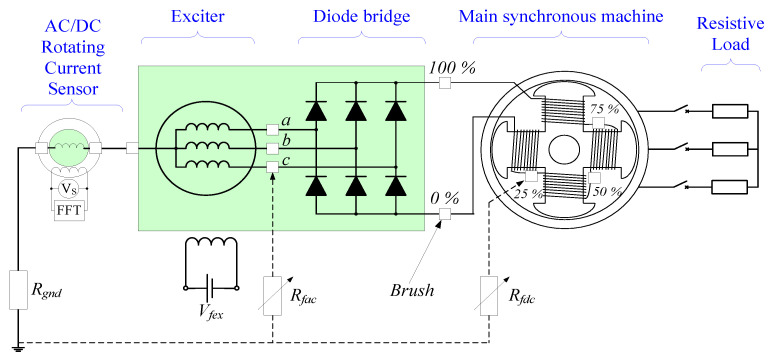
Experimental setup: simplified electrical diagram.

The experimental setup comprises three machines, as shown in Figure 6. A special BSM (1) has been used. This “brushless” machine comprises 6 auxiliary slip rings and brushes for metering purposes. The electrical diagram and the brushes’ location can be observed in Figure 6. The rotating machine is driven by an asynchronous motor (2). The BSM has a set of brushes and slip rings (3) connected to several points of the machine rotor. These points can be seen schematically in Figure 7. Therefore, it is possible to perform several internal faults and also to connect the neutral of the exciter to the rotating current sensor (4). The rotor winding of the rotating current sensor is grounded through a 5 kΩ limiting resistor, *R_gnd_*. This resistor limits the fault current to a low enough value that does not damage the equipment. The internal faults are performed by the use of a variable resistor (5), configurable from 0 Ω to 5 kΩ (*R_f_* = [0%, 100%] of *R_gnd_*). Also, the field winding of the exciter is fed by an adjustable voltage source (6). Finally, the sensor-induced voltage is recorded by a FLUKE 196B oscilloscope (7), where FFT is performed. The main characteristics of the experimental setup (main machine, exciter, and sensor) are collected in Appendix A.

**Figure 8:**
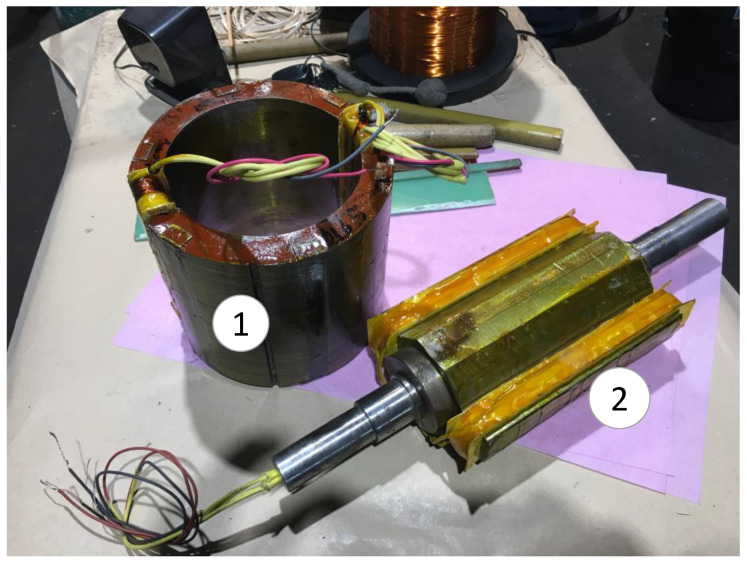
AC/DC rotating current sensor during manufacturing. [(1): stator; (2): rotor].

The sensor has been coupled to the shaft of the BSM through a flexible coupling. Both machines’ shafts were aligned by adjusting the position of the sensor with shims. The slip rings of the sensor have been connected to the slip rings of the BSM, as shown in the electrical scheme of the experimental setup (Figure 7). Furthermore, the rotating current sensor geometry can be seen in Figure 8, where the stator and rotor can be observed before their placement inside the sensor frame.

Regarding the different operation frequencies, the main machine has 2 pair of poles, which implies that the shaft rotates at *f_r_* = 25 Hz. However, the exciter has 6 pair of poles, which indicates that the currents flowing through the armature winding have a frequency of *f_exc_* = 150 Hz.

## 4. Experimental Results

Numerous experimental tests have been performed in the experimental setup to corroborate the operation of the proposed ground fault detection methodology.

### 4.1. Healthy Conditions Tests

The first set of the tests involved the healthy conditions. For this purpose, the main machine was operated first at no-load conditions and then fed a resistive load. The no-load test was accomplished at a rated voltage. Afterward, the resistive load was connected to the stator of the main machine. As the resistive load is not adjustable, the excitation current of the exciter, *I_exc_*, was modified to vary the current supplied by the main machine armature. Consequently, the armature voltage changes accordingly.

**Figure 9:**
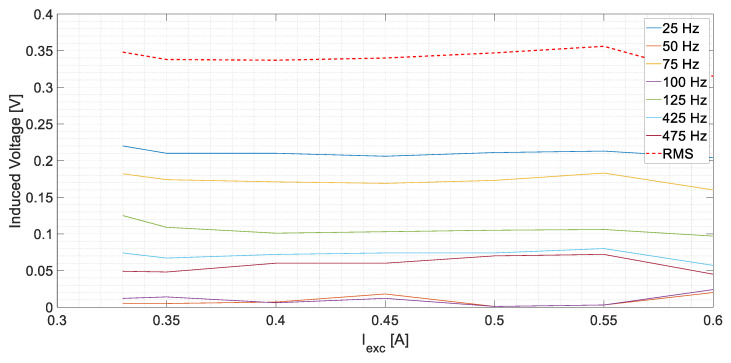
*U_S_* measurements corresponding to healthy conditions.

As can be clearly observed in Table 1, the induced voltages in the stator of the current sensor are almost constant and below 0.360 V for any operating condition. The results are also represented in Figure 9. This happens because the current that returns through the neutral of the exciter is negligible. Only parasitic capacitive currents would flow through the sensor. Then, the detection method is not altered due to load changes, which is a clear advantage.

### 4.2. Rotor DC Side Ground Faults: Main Machine Excitation Winding

After verifying that the current sensor is not affected by load changes during healthy operation, the first ground faults were implemented in the field winding of the main machine (see F1 position in Figure 4). The fault resistance *R_f_* varied from 5 kΩ to 0 kΩ to emulate different fault severities. Moreover, the faults were located at the 0%, 25%, 50%, 75%, and 100% of the field winding length. The negative terminal and the positive terminal of the winding correspond to 0% and 100%, respectively (See Figure 7).

**Figure 10:**
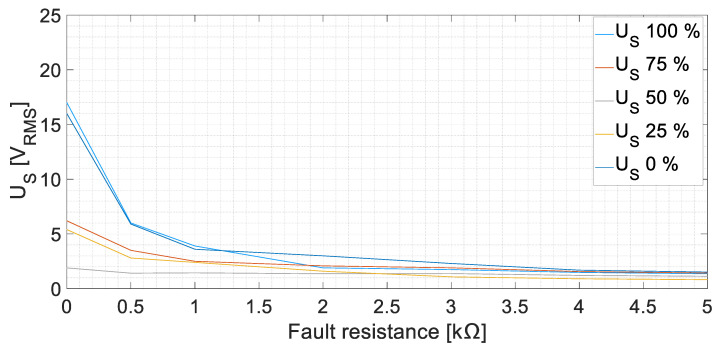
Sensor-induced voltage, *U_S_*, RMS values for GF in the field winding of the main machine at no-load conditions [fault positions 0, 25, 50, 75, and 100%].

The root mean square (RMS) values of the sensor-induced voltage, *U_S_*, are displayed in Figure 10 at no-load conditions. The induced voltage increases as the fault resistance decreases. In the case of *R_f_* = 5 kΩ faults, the voltages in the armature winding of the sensor are between 0.82 V and 1.52 V; meanwhile, for zero-resistance faults, the values increase considerably, to between 1.90 V and 17 V. Therefore, a ground fault can be easily detected as the voltage increases almost by three times in the worst case (*R_f_* = 5 kΩ). However, the faults in the midpoint of the windings apparently induce less voltage than the faults in the ends of the winding.

**Figure 11:**
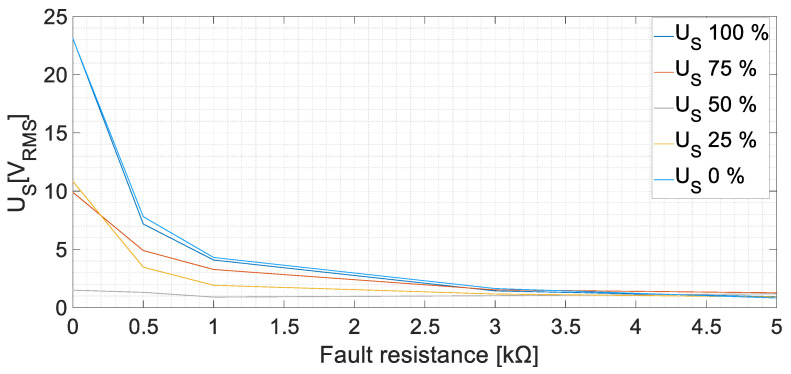
Sensor-induced voltage, *U_S_*, RMS values for GF in the field winding of the main machine at rated voltage on load conditions [fault positions 0, 25, 50, 75, and 100%, *I_Load_* = 8.1 A].

The tests were repeated with different armature currents changing the resistive load. The results are similar to those at no-load. In Figure 11, the results, corresponding to an armature current of 8.1 A at a rated voltage, are presented as an example. The induced voltage increases as the fault severity level increases. A 5 kΩ fault can be easily detected, as the induced voltage increases between 1.16 V and 1.79 V.

**Figure 12:**
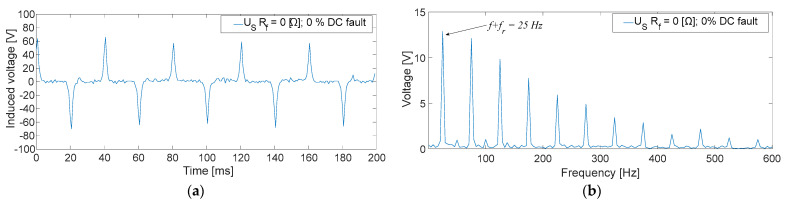
*U_S_* measurements corresponding to a GF in the field winding of the main machine at no-load conditions [fault position: 0%; *R_f_* = 0 [Ω]; (**a**): time domain; (**b**) frequency domain].

As an example, Figure 12a collects a 200 ms time domain measurement of the induced voltage in the sensor. The fault current that circulates through its rotor windings is mostly DC because the fault has been performed in the 0% of the main machine exciter winding with *R_f_* = 0 Ω. Therefore, the induced voltage will present narrow peaks due to the built sensor characteristics.

In this case, observing Figure 12b, the fundamental frequency when FFT is performed is 25 Hz. This corresponds with the fault current frequency added to the rotating frequency (*f* + *f_r_*).

### 4.3. Rotor AC Side Ground Faults: Armature Exciter Winding

**Figure 13:**
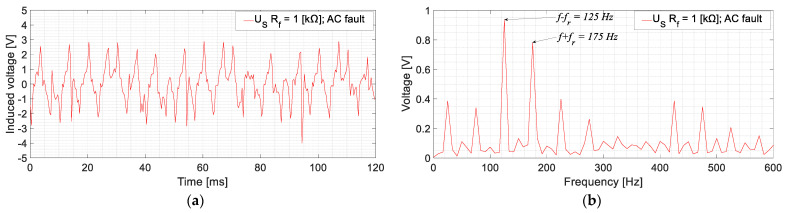
*U_S_* measurement for a GF in the armature winding of the exciter for no-load conditions [*R_f_* = 1 kΩ; (**a**): time domain; (**b**) frequency domain].

Furthermore, different ground faults have been accomplished in the AC side of the rotor circuit, corresponding to the armature windings of the exciter. This type of fault implies fault currents with 150 Hz frequency, i.e., the frequency of the induced currents in the armature windings of the exciter. Then, the frequencies induced in the stator of the sensor are *f* − *f_r_* = 125 Hz and *f* + *f_r_* = 175 Hz, as it can be seen in Figure 13. This figure shows an example of AC fault with *R_f_* = 1 kΩ.

**Figure 14:**
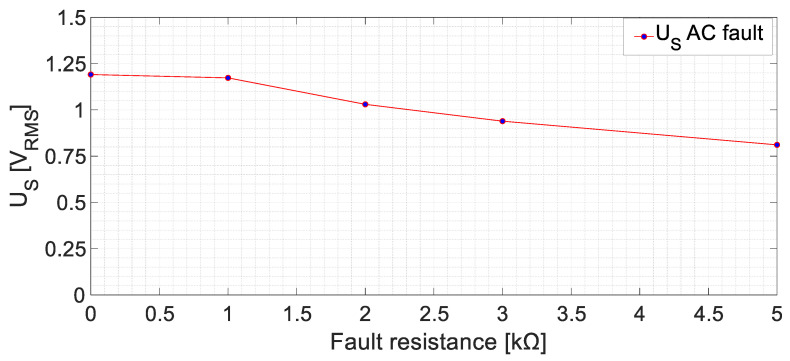
Sensor-induced voltage (RMS), *U_S_*, for ground faults in the armature winding of the exciter machine at no-load conditions.

Additionally, the results of the AC side fault tests with different *R_f_* are shown in Figure 14. The AC side faults can be detected as the RMS voltage increases from 0.36 V (healthy state) to 0.80 V (an increase of 222.3% in the RMS value for the *R_f_* = 5 kΩ case).

## 5. Discussion

Based on the experimental results shown in the previous section, several remarks can be made.

First of all, it must be highlighted that the results at healthy conditions are not affected by load changes. However, the *U_S_* measured in the stator of the current sensor was not null. This is because, even with no ground fault, parasitic currents will flow through *R_gnd_*. The maximum *U_S_* value achieved was 0.356 V_RMS_.

On the other hand, thanks to the high value of *R_gnd_*, the leakage current is limited to 10 mA, allowing for a continuous operation during the first ground fault. Two types of faults have been performed: in the field winding of the main machine and in the armature winding of the exciter.

The faults in the excitation winding of the main machine (DC faults) presented a higher peak in the FFT of *U_S_* in the 25 Hz harmonic. This harmonic corresponds to the DC component added to the rotor frequency. On the one hand, the effect of the position was explored, observing that the faults in the midpoint of the winding presented considerably lower RMS values than the faults in the terminals of the winding. This is due to the system symmetry [18]. On the other hand, the fault resistance change explored up to 100% of the value of *R_gnd_*. Observing Figure 10, when *R_f_* is higher (lower fault severity), the sensor stator voltage decreases. The behavior is similar to a voltage divider between *R_gnd_* and *R_f_*. Analogous results have been achieved in other works for different *R_f_* faults [26]. Another remark should be focused on the rectifier commutation frequency 3·*f_exc_* = 450 Hz. According to Section 2, the frequencies 425 Hz and 475 Hz were expected to appear when performing faults in the DC side, as the voltage ripple should be reflected in the fault current. However, those components reach low values. The explanation for this fact is that the inductive sensor actuates as a low-frequency filter, adding impedance to the fault circuit path at higher frequencies. Therefore, the fault current becomes even more limited and, as a consequence, the induced voltage in the stator becomes lower. Despite this filtering effect, faults in the midpoint of the winding can be observed thanks to the homopolar currents (450 Hz) returning through *R_gnd_* and the current sensor. The lowest RMS value of *U_S_* in the DC cases is 0.82 V, which means more than twice the value measured during healthy conditions.

Faults in the armature winding of the exciter (AC side) presented *U_S_* higher harmonics at 125 Hz and 175 Hz, which mainly correspond to 150 Hz (armature current frequencies) ±25 Hz (rotating frequency), according to the Leblanc theorem. In this case, only the fault resistance behavior was analyzed. As in the experimental setup, no intermediate positions could have been accessed without damaging the equipment’s insulation. As seen in Figure 13, the RMS values are considerably lower than for DC faults. This is due to the filtering effects of the sensor. As previously commented, now the fault currents are mainly 150 Hz instead of 0 Hz; then, the inductance of the sensor limits even more the fault current. In any case, the lowest *U_S_* RMS value, corresponding to a *R_f_* = 5 kΩ fault, is 0.80 V. Again, this value is greater than two times the value measured in healthy conditions.

Thus, it can be stated that the detection of ground faults in the rotor of BSMs can be performed by installing a rotating current sensor between the neutral of the armature current winding and the rotor’s shaft and analyzing the frequency spectrum of its induced voltage.

## 6. Conclusions

Rotor measurements are difficult in brushless machines. This makes considerably difficult the protection and monitoring of the internal rotor parts. This paper has proposed a ground fault detection method for the rotor of BSMs based on using an AC/DC rotating current sensor.

The method proposes the coupling of a rotating AC/DC current sensor between the neutral of the exciter armature winding and the shaft in series with a resistive current limiter. Thus, if a ground fault happens in the rotor, the fault circuit path is closed through the rotor shaft and passes through the sensor. This current induces a voltage in the sensor’s stator where a frequency analysis is performed. Observing the induced voltage, the fault can be detected due to a large increase in its RMS value. For example, in the tested machine, the maximum RMS-induced voltage is 0.348 V in healthy conditions. The ground fault that causes the minimum induced RMS voltage corresponds to a ground fault in the rotor midpoint. In the case of a 5 kΩ ground fault at that point, the obtained RMS voltage is 1.16 V, i.e., three times the induced voltage in healthy conditions. For an industrial synchronous machine, checking the induced RMS voltage in healthy conditions at no-load and full load would be needed. To adjust the threshold of the protection, making a 5 kΩ ground fault and measuring the RMS-induced voltage would also be needed. In the case that this ground fault could not be performed, a reasonable setting could be three times the RMS voltage in healthy conditions.

In the case of a ground fault, the different harmonics can help to locate the fault as the zone can be discerned between the armature winding faults (AC side) or the main machine excitation winding faults (DC side), where DC faults present their main harmonic at the rotor frequency and AC faults present their main harmonics at the armature current frequency plus/minus the rotor frequency.

The proposed detection method has been checked using experimental tests, obtaining results according to the previous formulated hypotheses.

In such a manner, this method can detect faults up to 5 kΩ (maximum value tested in the laboratory), which is a normal setting of existing rotor ground fault protections. The measured RMS value increases more than three times compared with healthy conditions. In addition, the fault zone, AC or DC, can be discerned. However, its main weakness appears when analyzing higher frequencies, i.e., as the current sensor performs as a low-frequency filter, the fault current sensitivity for high frequencies is reduced.

Based on the results obtained in this research, further works should focus on two fields: On the one hand, the fault location problem remains unsolved. On the other hand, modified current sensor designs could achieve better results and sensitivities.

## 7. Patents

The Spanish patent ES 2798048 B2 (4/12/2020) “Rotating Sensor for the Altern and/or Direct Current Measurement in Rotating Machines” was developed and is considered in the development of this research work to be authorized by M. A. Pardo-Vicente, C. A. Platero, F. Blázquez, and J. A. Sánchez.

## Figures and Tables

**Table 1:** Healthy conditions experimental test results.

Operation Conditions			Current Sensor-Induced Voltage *U_S_* [V]							
*I_exc_* [A]	*V_stator_* [V]	*I_stator_* [A]	25 [Hz]	50 [Hz]	75 [Hz]	100 [Hz]	125 [Hz]	425 [Hz]	475 [Hz]	RMS [V]
0.33	384	0.0	0.220	0.005	0.182	0.012	0.125	0.074	0.049	0.348
0.35	274	5.9	0.210	0.005	0.174	0.014	0.109	0.067	0.048	0.338
0.40	296	6.4	0.210	0.007	0.171	0.006	0.101	0.072	0.060	0.337
0.45	320	6.8	0.206	0.018	0.169	0.012	0.103	0.074	0.060	0.340
0.50	340	7.3	0.211	0.001	0.173	0.001	0.105	0.074	0.070	0.347
0.55	362	7.7	0.213	0.003	0.183	0.003	0.106	0.080	0.072	0.356
0.60	382	8.1	0.204	0.020	0.160	0.024	0.097	0.057	0.045	0.315

## Data Availability

Data will be made available upon reasonable request.

## Appendix A

**Table A1:** Synchronous machine data.

Parameter	Magnitude	Units
Machine type	Brushless Synchronous Machine	
Rated power	12.5	kVA
Rated speed	1500	rpm
Rated voltage	400	V
Rated power factor	0.8	
Rated current	18	A
Phases	3	
Rated frequency	50	Hz
Isolation class	F	

**Table A2:** Exciter machine data.

Parameter	Magnitude	Units
Machine type	Synchronous brushless	
Rated speed	1500	rpm
Rated excitation voltage	15	V
Rated excitation current	1.5	A
No-load excitation voltage	4.25	V
No-load excitation current	0.5	A
Phases	3	
Number of poles	12	
Isolation class	F	

**Table A3:** Sensor data.

Parameter	Magnitude	Units
Machine type	Prototype sensor	
Number of field poles	2	
Number of armature poles	2	
Number of turns of field winding	1000	
Number of turns of armature winding	3000	
Wires section	0.096	mm^2^
Rated speed	1500	rpm
Rotor diameter	122.5	mm
Air gap	0.5	mm
